# Antimicrobial effect of farnesol, a *Candida albicans *quorum sensing molecule, on *Paracoccidioides brasiliensis *growth and morphogenesis

**DOI:** 10.1186/1476-0711-8-13

**Published:** 2009-04-29

**Authors:** Lorena S Derengowski, Calliandra De-Souza-Silva, Shélida V Braz, Thiago M Mello-De-Sousa, Sônia N Báo, Cynthia M Kyaw, Ildinete Silva-Pereira

**Affiliations:** 1 Laboratório de Biologia Molecular, CEL/IB, Universidade de Brasília – Brasília-DF, 70910-900, Brasil; 2 Laboratório de Microscopia Eletrônica, CEL/IB, Universidade de Brasília – Brasília-DF, 70910-900, Brasil; 3 Laboratório de Microbiologia, CEL/IB, Universidade de Brasília – Brasília-DF, 70910-900, Brasil

## Abstract

**Background:**

Farnesol is a sesquiterpene alcohol produced by many organisms, and also found in several essential oils. Its role as a quorum sensing molecule and as a virulence factor of *Candida albicans *has been well described. Studies revealed that farnesol affect the growth of a number of bacteria and fungi, pointing to a potential role as an antimicrobial agent.

**Methods:**

Growth assays of *Paracoccidioides brasiliensis *cells incubated in the presence of different concentrations of farnesol were performed by measuring the optical density of the cultures. The viability of fungal cells was determined by MTT assay and by counting the colony forming units, after each farnesol treatment. The effects of farnesol on *P. brasiliensis *dimorphism were also evaluated by optical microscopy. The ultrastructural morphology of farnesol-treated *P. brasiliensis *yeast cells was evaluated by transmission and scanning electron microscopy.

**Results:**

In this study, the effects of farnesol on *Paracoccidioides brasiliensis *growth and dimorphism were described. Concentrations of this isoprenoid ranging from 25 to 300 μM strongly inhibited *P. brasiliensis *growth. We have estimated that the MIC of farnesol for *P. brasiliensis *is 25 μM, while the MLC is around 30 μM. When employing levels which don't compromise cell viability (5 to 15 μM), it was shown that farnesol also affected the morphogenesis of this fungus. We observed about 60% of inhibition in hyphal development following *P. brasiliensis *yeast cells treatment with 15 μM of farnesol for 48 h. At these farnesol concentrations we also observed a significant hyphal shortening. Electron microscopy experiments showed that, despite of a remaining intact cell wall, *P. brasiliensis *cells treated with farnesol concentrations above 25 μM exhibited a fully cytoplasmic degeneration.

**Conclusion:**

Our data indicate that farnesol acts as a potent antimicrobial agent against *P. brasiliensis*. The fungicide activity of farnesol against this pathogen is probably associated to cytoplasmic degeneration. In concentrations that do not affect fungal viability, farnesol retards the germ-tube formation of *P. brasiliensis*, suggesting that the morphogenesis of this fungal is controlled by environmental conditions.

## Background

Essential oils are complex mixes of hydrophobic liquids containing volatile aromatic compounds, which are products of plant secondary metabolism [1]. Of all the claimed properties of essential oils, its antimicrobial activity is the one which receives special attention due to the serious threat that antibiotic resistance has become. Therefore, the study of potential antibiotic compounds found in these oils could be of interest in the development of novel antimicrobial agents.

Farnesol is a sesquiterpene alcohol present in many essential oils – e.g. from *Pluchea dioscoridis*, *Zea mays *and *Pittosporum undulatum*, possibly protecting these plants from parasitic induced damages [2-4]. Recently, this sesquiterpene alcohol has been demonstrated to inhibit the growth of some microorganisms, such as the human pathogens *Staphylococcus aureus *[5,6] and *Streptococcus mutans *[7], and the plant pathogenic fungus *Fusarium graminearum *[8], signaling its potential use as an antimicrobial agent. Farnesol also enhances microbial susceptibility to antibiotics, indicating a putative application as an adjuvant therapeutic agent [9,10]. Although its mechanism of action is not fully understood, it probably involves cell membrane damages and impaired ergosterol synthesis [10].

This sesquiterpenoid was also identified as a quorum-sensing molecule produced by the dimorphic fungus *Candida albicans*, where it prevents the fungal transition from yeast to mycelium, and disrupts biofilm formation [11,12]. *C. albicans *synthesizes farnesol from farnesyl pyrophosphate (FPP), a well known intermediate of the highly conserved sterol biosynthetic pathway [13]. A recent study showed that farnesol increases the virulence of *C. albicans *in a mouse infection model [14]. In another work, it appears that farnesol is employed by *C. albicans *in order to reduce competition with other microbes, since this compound mediated apoptosis in the filamentous fungus *Aspergillus nidulans *[15], and inhibited biofilm formation in other *Candida *species [10,16].

In this study, we tested the effects of farnesol on *Paracoccidioides brasiliensis *growth and morphogenesis. *P. brasiliensis *is the etiologic agent of paracoccidioidomycosis (PCM), a systemic human mycosis geographically confined to Latin America [17,18]. This organism is a thermal dimorphic fungus, which can be found as mycelium at room temperature (25°C) and as yeast cells at body temperature (37°C). Although little is known about the ecology of this fungus, it is thought that infection occurs when the mycelial form releases conidia or hyphal fragments to the environment and, upon inhalation by the host, these structures differentiate to the yeast form [19]. This dimorphic transition of mycelium to yeast phase seems to be essential to the establishment of the infective process [20]. In this context, our results revealed that farnesol reduces the viability of this pathogen and delays the dimorphism, suggesting an antimicrobial activity against *P. brasiliensis*, probably due the massive cytoplasmic organelles degeneration.

## Methods

### Fungal strain

Yeast cells of the virulent isolate 18 of *P. brasiliensis *(Pb18) were maintained by weekly passages in semi-solid Fava Neto's medium (0.3% protease peptone, 1% peptone, 0.5% beef extract, 0.5% yeast extract, 4% glucose, 0.5% NaCl, 1.6% agar, pH 7.2) at 36°C, and were used after 6 – 9 days of growth. The fungal cells used in all experiments were suspended in complex medium YPD (yeast-peptone-dextrose), vigorously vortexed and counted in a Neubauer chamber. The cell viability was determined by vital Janus green stain [21]. *C. albicans *ATCC 10231, used as a control, was also maintained on semi-solid Fava Neto's medium at 36°C and transferred at regular intervals. Under these conditions over 95% of *C. albicans *cells remained in the yeast form.

### *P. brasiliensis *growth assay upon farnesol treatment

In all assays, a mixture of stereoisomers of farnesol (assay ≥ 90%; GS, sum of isomers; Fluka, Sigma-Aldrich) was diluted in 100% methanol. Working concentrations were prepared in YPD medium.

For the *in vitro *growth assay, 2 × 10^5 ^*P. brasiliensis *yeast cells per mL were inoculated in complex medium YPD supplemented with farnesol at different final concentrations (5, 10, 25, 50, 100, 150 and 300 μM), employing 96 well-plates in a final volume of 200 μL. Farnesol-free controls were supplemented with 2% methanol, the farnesol diluent. Cultures were allowed to grow at 36°C for 25 days. The number of cells at each specific time interval was determined by measuring the OD absorption at 630 nm of vigorously vortexed cultures. The growth curve of each culture was prepared by plotting the logarithmic values of OD630 *vs*. incubation time. All experiments were carried out in triplicate. The Minimum Inhibitory Concentration (MIC) for farnesol was defined as the lowest concentration that resulted in 90% of inhibition of cell growth when compared to the farnesol-free control cells.

### Effect of farnesol on *P. brasiliensis *cell viability

The analysis of cell viability was performed using the tetrazolium salt 3-[4,5-dimethylthiazol-2-yl]-2,5-diphenyltetrazolium bromide (MTT; Sigma) in a colorimetric assay that measures mitochondrial activity. To determine the MLC (Minimal Lethal Concentration) of farnesol for *P. brasiliensis*, 2 × 10^5 ^yeast cells/mL were incubated in YPD medium with farnesol at the final concentrations of 2.5, 5, 7.5, 10, 12.5, 15, 17.5, 20, 22.5, 25, 27.5, 30, 50, 75, 100, 150 and 300 μM. After 15 days of incubation with different concentrations of farnesol at 36°C (150 rpm) in 96-well tissue culture plates, a solution containing 5 mg of MTT per mL of 0.15 M phosphate-buffered saline (PBS) was added to each well to reach a final concentration of 0.5 mg/mL. After incubation for 4 h at 36°C, the medium containing MTT was partially removed, and dimethyl sulfoxide (100 μL) was added to solubilize the MTT formazan product. MTT formazan formation was measured at 490 nm by using a spectrophotometer. Control wells contained medium plus MTT to determine background formazan values. All assays were done in triplicate. The MLC for farnesol was defined as the lowest concentration that resulted in 90% of cell death when compared to the farnesol-free control cells.

The effect of farnesol on cell viability was also estimated by colony counting. Yeast cells of *P. brasiliensis *were suspended in YPD medium at a density of 2 × 10^5 ^cells/mL, and farnesol was added to final concentrations of 5, 10, 25, 50 and 100 μM. Farnesol-free controls were supplemented with 2% methanol. After incubation at 36°C for 4 days in an orbital shaker (150 rpm), cells were harvested by centrifugation (4000 × g/5 min), washed and plated on brain heart infusion agar (BHI) supplemented with 4% fetal calf serum (FCS), for at least 12 days to determine their viability, expressed as colony forming units (CFU). The survival rate of these cultures was compared to farnesol-free cultures. The number of single colonies on each plate was counted and the percentage of cell killing calculated as (1 - N1/N2) × 100, where N1 is the mean of the number of colonies from farnesol-treated *P. brasiliensis *cells, and N2 is the mean of the number of colonies from *P. brasiliensis *non-treated cells. The experiments were carried out in triplicate.

### Effect of farnesol on *P. brasiliensis *dimorphic transition

To evaluate the effects of farnesol on *P. brasiliensis *dimorphism, 2 × 10^5 ^yeast cells/mL were inoculated in YPD medium and farnesol was added to final concentrations of 5, 7.5, 10, 15, 20 and 25 μM. Cultures were incubated for 48 h at 25°C in an orbital shaker (150 rpm). Farnesol-free controls were supplemented with 2% methanol, the farnesol diluent. Cell morphology was assessed by light microscopy. The percentage of cells showing germ-tubes after 24 h and 48 h of incubation was calculated. Additionally, the average size of the germ tubes was measured after the same period of time. All experiments were carried out in triplicate. The *P. brasiliensis *dimorphism from mycelium to yeast cells was also evaluated by the treatment of the mycelial form with farnesol at different final concentrations (5, 10, and 25 μM), for 4 days at 36°C, following exactly the same conditions described above for the transition in the opposite direction.

### Transmission and Scanning electron microscopy

Transmission electron microscopy (TEM) was performed according to the following standard procedure. Briefly, *P. brasiliensis *yeast cells were cultivated at 36°C for 3 days in YPD medium without (control supplemented with 2% methanol) or with farnesol (25, 50 and 150 μM). Cells were harvested by centrifugation at 4000 × g for 5 min, washed four times with phosphate-buffered saline (PBS, pH 7.2) and fixed overnight at 4°C (2% glutaraldehyde, 2% paraformaldehyde in 0.1 M sodium cacodylate buffer, pH 7.2, with 3% sucrose and 3 mM CaCl_2_). After fixation, cells were harvested by centrifugation (4000 × g/5 min), and the pellet was washed four times in 0.1 M sodium cacodylate buffer (4000 × g/15 min). Samples were post-fixed for 1 hour (1% osmium tetroxide, 0.8% potassium ferrocyanide in the same buffer), contrasted *en bloc *with 0.5% uranyl acetate, dehydrated through an ascending acetone series and embedded in Spurr resin. The ultrathin sections were contrasted with uranyl acetate/lead citrate and observed in a TEM Jeol 1011 at 80 kV.

In order to prepare samples to scanning electron microscopy analysis, *P. brasiliensis *yeast cells treated with different concentrations of farnesol (25 and 50 μM) for 5, 10 and 24 h, were fixed overnight at 4°C (2% glutaraldehyde, 2% paraformaldehyde in 0.1 M sodium cacodylate buffer, pH 7.2, with 3% sucrose and 3 mM CaCl_2_), harvested by centrifugation (4000 × g/5 min), and the pellet was washed four times in 0.1 M sodium cacodylate buffer (4000 × g/15 min). Samples were post-fixed for 1 hour (1% osmium tetroxide, 0.8% potassium ferrocyanide in the same buffer), and then applied on a polylysine-coated coverslip and serially dehydrated in acetone. The samples were dried in a critical point drier (BAL-TEC CPD-030 – Electron Microscopy Sciences, USA), coated with gold-palladium (Balzers Union SCD-040 – Electron Microscopy Sciences, USA) and viewed using a JEOL (Tokyo, Japan) JEM 840A electron microscope.

### Statistical analysis

Statistical analyses were performed using the software "Mynova", verson 1.3 (S. Brooks, Copyright 1993). The statistical test applied was Student's *t *test. A *P *value ≤ 0.001 was considered significant.

## Results and discussion

### Farnesol affects *P. brasiliensis *growth and viability

The incidence of fungal infections has dramatically increased in the last two decades [22,23]. Simultaneously, the resistance to antifungal agents has become an important problem in several fungal diseases. In addition to the drug resistance problem, the current antifungal therapies are limited due the high toxicity of the agents and their low efficacy rates [24].

PCM is commonly treated with sulfonamides, azoles and amphotericin B [25]. In spite of the good efficacy of these antifungal agents against *P. brasiliensis*, some of these compounds, like amphotericin B, can also damage the host cells [26]. Furthermore, Hanh et al. [27] described the occurrence of ketoconazole resistant isolates of *P. brasiliensis *in PCM patients. The *P. brasiliensis *transcriptome analysis revealed several ortholog genes related to transmembrane proteins that can function as efflux pumps [28]. The existence of such genes augurs the possible emergency of resistant isolates, emphasizing the importance of novel antimicrobial agent development.

In this context, the interest in studying the antimicrobial activity of plant extracts and essential oils has increased in recent years. San-Blas et al. [29] demonstrated that ajoene, a compound from *Allium sativum*, inhibits the growth of *P. brasiliensis*. These authors suggested that the integrity of the fungal cytoplasmic membrane could probably be the target of this garlic-derived compound, since ajoene promotes changes in the phospholipid and fatty acid proportions [30]. Furthermore, the association of ajoene and chemotherapeutic drugs show a positive additive effect in the treatment of mice infected with *P. brasiliensis *[31].

In this work we evaluated the role of farnesol, a sesquiterpene alcohol present in many essential oils [2-4,32], and also produced as a quorum sensing molecule by *C. albicans*, in *P. brasiliensis *growth and morphogenesis.

**Figure 1:**
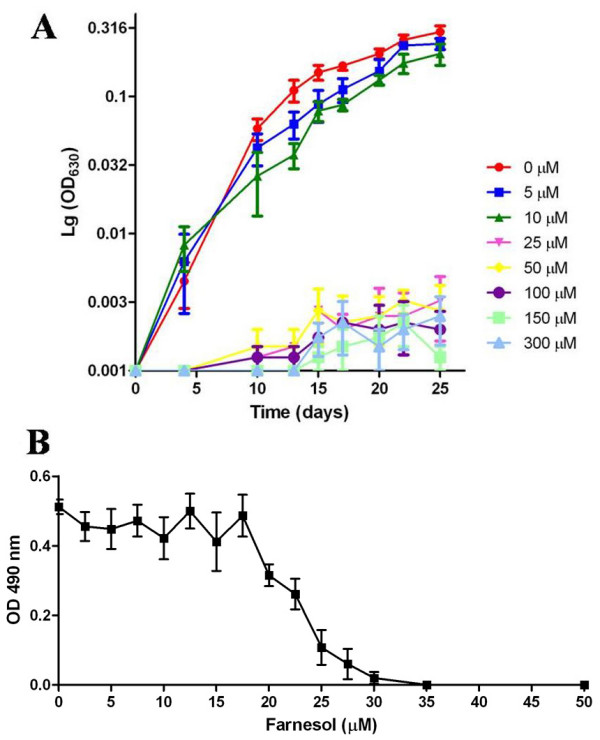
**Effect of farnesol on the growth and viability of *P. brasiliensis *yeast cells**. (A) Growth curves of *P. brasiliensis *yeast cells incubated in the presence of different concentrations of farnesol. The number of cells at each specific time point was assessed by measuring the OD absorption at 630 nm, after vigorously shaking the cultures. The growth curve of each culture was prepared by plotting the logarithmic values of OD630 *vs*. incubation time. (B) Viability of *P. brasiliensis *yeast cells cultivated with different concentrations of farnesol after 15 days of growth in the absence, or in the presence of farnesol (2.5 to 50 μM). The MTT assay was performed as described in methods section. MTT formazan formation was measured at 490 nm by using a spectrophotometer.

First, the effect of farnesol on *P. brasiliensis *growth was evaluated. We verified that farnesol concentrations ranging from 25 to 300 μM strongly inhibited *P. brasiliensis *yeast cells growth, since the results obtained with three different growth curves were equivalent (Figure 1A). Similar patterns were observed with the mycelial form growth curves (data not shown).

In order to evaluate if the effects of farnesol on *P. brasiliensis *growth were caused by an increase in mortality, a viability assay based on metabolism (Figure 1B) was performed (MTT assay). This test showed that the percentage of non-viable fungal cells increased proportionally with farnesol concentrations, suggesting that farnesol has a potent fungicide activity against *P. brasiliensis*. The same behavior of farnesol on *P. brasiliensis *viability was observed when we performed experiments based on CFU counts (data not shown). The Figure 1A reveals that farnesol concentrations of 25 μM or higher have an inhibitory effect on *P. brasiliensis *growth, while *P. brasiliensis *metabolism completely ceased at the farnesol concentration of 30 μM (Figure 1B). According to these data we have determined that the MIC of farnesol for *P. brasiliensis *is 25 μM, while the MLC is 30 μM.

This role of farnesol in the inhibition of *P. brasiliensis *growth and viability has also been verified in other microorganisms [6-8,33-36], suggesting that this compound also possess an effective antimicrobial activity against *P. brasiliensis*. Interestingly, our data indicate that *P. brasiliensis *is more sensitive to farnesol than other pathogens since, while for *P. brasiliensis *the MLC corresponds to 30 μM, the MLC is of 200 μM for the bacteria *S. aureus *and *Streptomyces tendae *[6,33]. The bacterium *S. mutans *and the fungus *Candida dubliniensis *show higher tolerances to farnesol, with an MLC of 300 μM and 500 μM, respectively [7,10].

### Farnesol delays the dimorphic transition of *P. brasiliensis*

**Figure 2:**
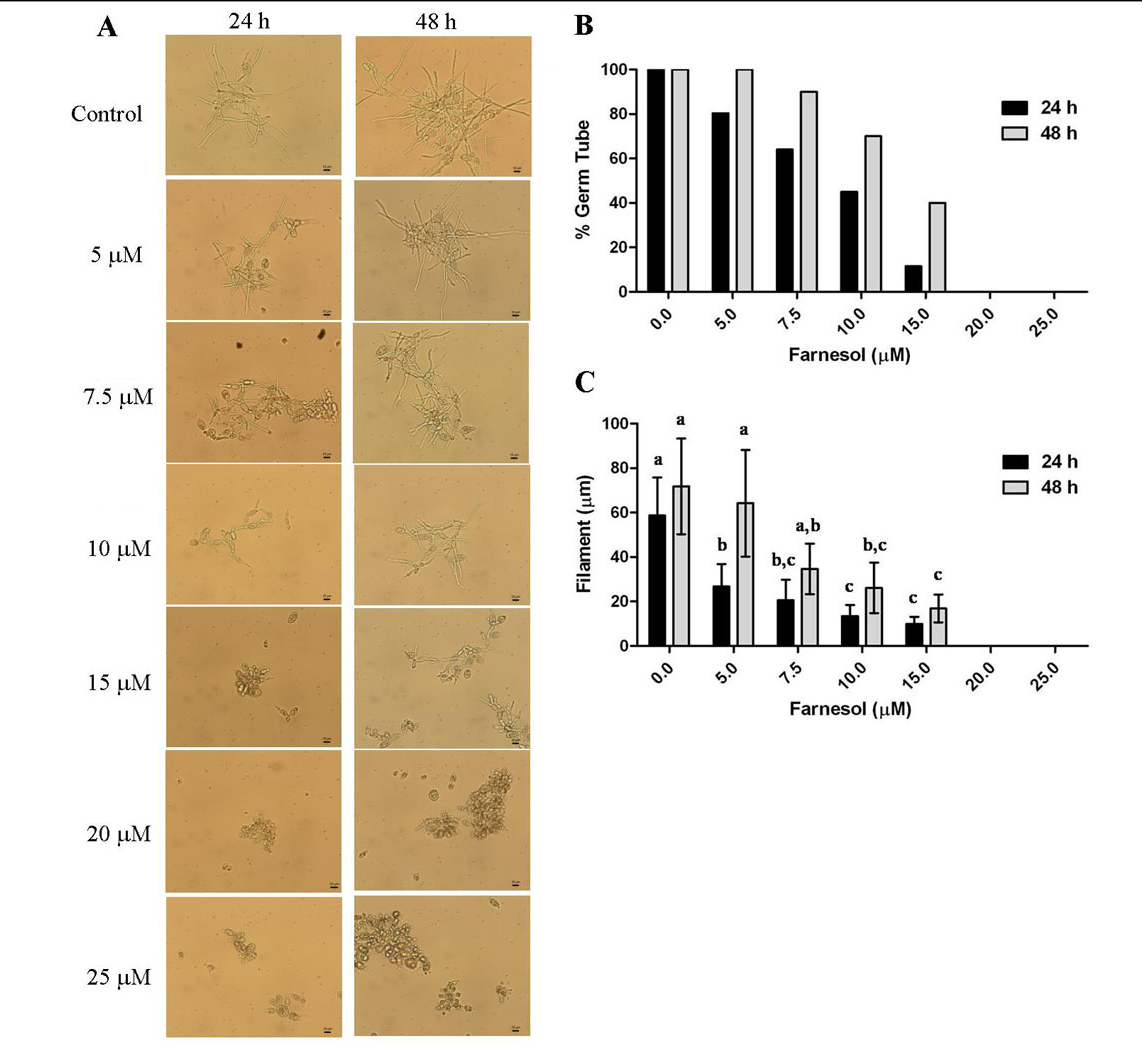
**Effect of farnesol on *P. brasiliensis *morphogenesis**. *P. brasiliensis *yeast cells were grown for 24 h and 48 h at 25°C on different concentrations of farnesol. (A) Morphology of *P. brasiliensis *cells assessed by light microscopy after incubation with farnesol. (B) Percentage of cells showing germ tubes after 24 h and 48 h of cultivation in the absence, or in the presence of farnesol at different final concentrations. (C) Average size of the germ tubes formed in different concentrations of farnesol. Bars represent standard errors and different letters point to statistical relevance, *P *≤ 0.001.

We have tested the effects of farnesol on *P. brasiliensis *morphogenesis employing farnesol concentrations up to 25 μM, the MIC value determined for *P. brasiliensis *in this study. Noteworthy, our results revealed that the addition of exogenous farnesol at concentrations below 25 μM, which do not affect *P. brasiliensis *growth, impaired the transition from yeast to mycelium, as observed by microscopic analyses of cellular morphology after 24 h and 48 h incubation (Figure 2A). When grown in the absence of farnesol, all cells presented germ tubes after 24 h, while when cultured in the presence of farnesol (up to 25 μM) we observed an increasingly lower percentage of cells with germ tubes after 24 h, with about 85% of cells without germ tubes at 15 μM of farnesol (Figure 2B). In addition, after 24 h, the germ tubes of cells treated with up to 15 μM of farnesol were markedly smaller when compared to those of control cells (Figure 2C). Figures 2B and 2C also reveal that, up to15 μM, farnesol is acting on the morphogenesis instead of the viability of *P. brasiliensis *cells since after 48 h of incubation we can observe an increase in both, the number of cells with germ tubes – 60% of cell with germ tubes at 15 μM of farnesol (Figure 2B), and in the size of these structures (Figure 2C). These data strongly suggest that in fact the farnesol at concentrations below 15 μM present an effect on the morphogenetic process of *P. brasiliensis *without interfering with its viability, as showed in figure 1B.

**Figure 3:**
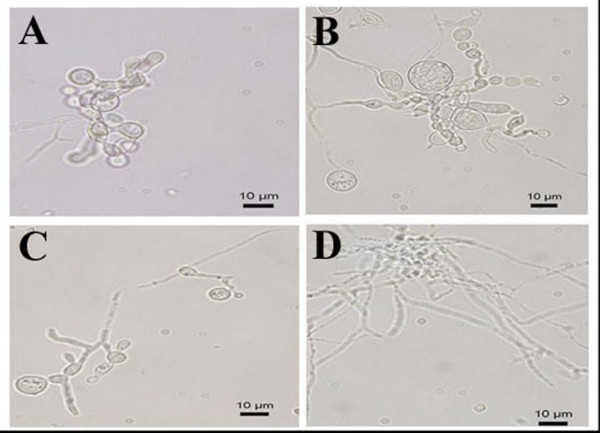
**Effects of farnesol on *P. brasiliensis *mycelium to yeast transition**. *P. brasiliensis *(mycelium form) was incubated for 4 days at 36°C on different concentrations of farnesol. (A) Control (no farnesol); (B) 5 μM farnesol; (C) 10 μM farnesol; (D) 25 μM farnesol.

Similar concentrations of this isoprenoid also inhibited *P. brasiliensis *mycelium to yeast transition, as verified after 4 days incubation of the mycelial form at 36°C (Figure 3). The cultivation of *P. brasiliensis *in the presence of 5 and 10 μM of farnesol resulted in the impairment of the dimorphic transition, while, as expected, 25 μM of farnesol completely abolished the fungal dimorphic transition, probably by compromising cell viability.

Similar results were observed with *C. albicans *cells, where farnesol also prevented germ tubes formation [11,37]. Other studies revealed that farnesol is also employed by *C. albicans *to mediate antagonistic interactions with other microorganisms [10,15,16]. Semighini et al. [15] showed that exogenous farnesol triggers apoptosis in the filamentous fungus *A. nidulans*. Moreover, the possibility that farnesol produced by *C. albicans *might affect *A. nidulans *was evaluated in a co-culture model, showing that the co-cultivation inhibits the growth of *A. nidulans *in a farnesol-dependent manner [15]. A recent work reported that the addition of farnesol to cultures of *Pseudomonas aeruginosa *leads to a decrease in the amount of *Pseudomonas *quinolone signal (PQS), and of the virulence factor pyocyanin produced by this pathogen. In addition, pyocyanin and PQS levels in *P. aeruginosa-C. albicans *co-cultures were reduced to 42.1% relative to a control pure culture of *P. aeruginosa*. A similar decrease was verified in farnesol-treated *P. aeruginosa *cultures, suggesting that farnesol may be involved in inter-kingdom interactions [38].

Noteworthy was the inhibition of *P. brasiliensis *hyphal development when yeast cells were grown on a conditioned medium (CM), which corresponds to the filtered supernatant of a high-density *C. albicans *culture added to a conventional culture medium (data not shown). This result suggests that *P. brasiliensis *germ tube formation could be controlled by a soluble factor present in the *C. albicans *culture supernatant [39]. In this sense, we propose that the farnesol-mediated communication between *C. albicans *and other microorganisms is probably not species-specific.

Curiously, tyrosol, another compound also identified as a quorum-sensing molecule produced by *C. albicans *[40], did not affect either *P. brasiliensis *growth or morphogenesis at concentrations up to 300 μM (data not shown).

### Ultrastructural morphology analysis of *P. brasiliensis *yeast cells treated with farnesol

Although the role of farnesol as a potential antimicrobial agent has been determined in this work, its mode of action is not understood. Some studies have indicated a possible interaction of farnesol with cell membranes of certain microorganisms, including the bacteria *S. mutans*, *S. aureus *and *E. coli*, [5-7,9,41], and the fungi *C. albicans *and *C. dubliniensis *[10]. A hypothesis is that the hydrophobic property of farnesol favors its accumulation in the membrane, causing membrane disruption [6].

**Figure 4:**
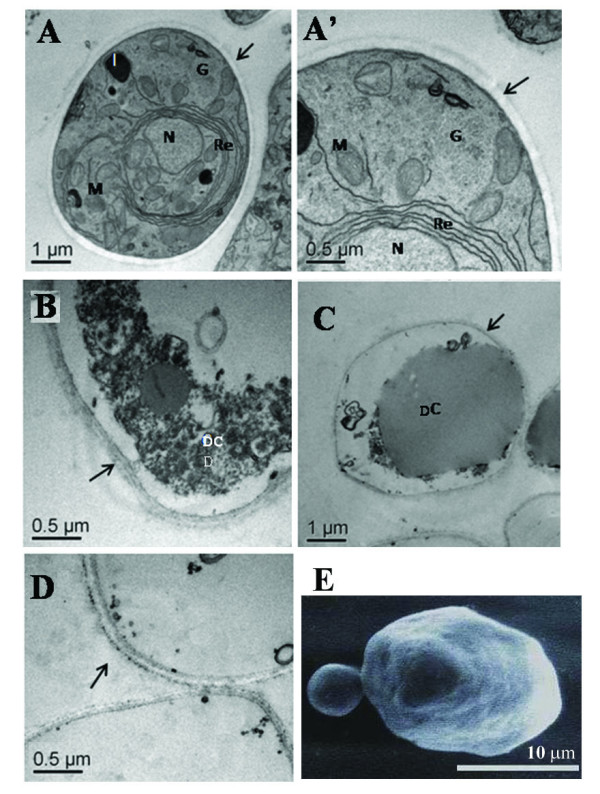
**Ultrastructural morphology of *P. brasiliensis *yeast cells treated with different concentrations of farnesol**. Pannels A to D show the transmission electron micrographs of cells cultivated in the absence (A, A') and in the presence of farnesol (B to D). Cell structures like nucleus (N), mitochondria (M), endoplasmatic reticulum (Re), lisossome-like structures (l), plasma membrane and cell wall are preserved in control cells (arrows in A, A'). Cells cultivated in 25 μM (B), 50 μM (C) and 100 μM (D) of farnesol showed a degraded cytoplasm (DC) but an intact cell wall (arrows). In panel E, the scanning electron micrograph of 50 μM farnesol-treated yeast cells of *P. brasiliensis *is shown, revealing an intact fungal cell wall.

In order to examine the effects of farnesol on the morphology of *P. brasiliensis *at the ultrastructural level, yeast cells were cultivated for three days on different concentrations of farnesol (25, 50 and 150 μM) and examined by transmission and scanning electron microscopy. As observed in figure 4A and 4A', *P. brasiliensis *yeast cells cultivated in the absence of farnesol (control cells supplemented with 2% of methanol) showed typical cellular structures. Cytoplasmic organelles of *P. brasiliensis *cells, such as mitochondria, nucleus, lysosomes, and endoplasmatic reticulum could be clearly distinguished. Cell wall and plasma membrane were also observed as intact structures (Figure 4A and 4A'). These observations clearly demonstrated that the use of 2% of methanol in all experimental systems as farnesol's diluent, did not affect *P. brasiliensis *cell morphology as well as growth and dimorphic transition.

In contrast, yeast cells treated with increasing concentrations of farnesol exhibited extensive cytoplasmic organelles damages. Remarkable changes, resulting in overall degeneration of internal structures, were found in *P. brasiliensis *cells cultivated in the presence of 25, 50 and 100 μM of farnesol (Figure 4B–D). Various stages of degradation were observed, ranging from cells with partially digested cytoplasmic organelles, to cells with only the cell wall remaining intact (Figure 4D). These results suggest that death is probably associated to the disruption of cytoplasmic structures and internal cellular disintegration.

Scanning electron microscopy was also performed employing *P. brasiliensis *yeast cells treated with farnesol, as described in material and methods. Cells treated with different concentrations of farnesol showed no major differences at their surfaces when compared to the control cells incubated without farnesol. Figure 4E shows the scanning electron micrograph of the 50 μM farnesol-treated yeast cells of *P. brasiliensis*, revealing an intact fungal cell. This result corroborates the data obtained by transmission electron microscopy, suggesting that farnesol does not affect the cell wall structure.

Similar results were reported by San-Blas et al. [29] studying the antifungal activity of ajoene, which blocks the growth of *P. brasiliensis *by inhibiting phosphatidylcholine synthesis [30]. Curiously, farnesol induces apoptosis in tumorigenic cells by a similar mode of action [42,43], suggesting an antiproliferative mechanism shared by ajoene and farnesol.

Moreover, our findings show that the fungicidal mechanism of farnesol is probably associated to the disruption of all cytoplasmic cellular organelles. A similar cytoplasm disintegration also occurs after the ingestion of *P. brasiliensis *yeast cells by cytokine-activated murine macrophages [44], indicating that this is possibly a common event in response to a stress condition.

In order to understand the mode of action of farnesol on *P. brasiliensis*, a microarray large scale analysis of gene expression, as well as assays to evaluate the cell death pathway activated in response to farnesol, should be performed. Of major interest is the analysis of the expression of genes related to phospholid and sterol synthesis pathways, as well and those related to the apoptotic process, which are in progress.

## Conclusion

In summary, our data indicate that farnesol acts as a potent antimicrobial agent against *P. brasiliensis*, which is very sensitive to this sesquiterpene alcohol. The fungicide activity of farnesol in this pathogen is probably associated to cytoplasmic degeneration, in spite of the apparent cell wall integrity, as observed by transmission and scanning electron micrographs. Although the antimicrobial activity of farnesol has been clearly shown, additional studies involving animal models need to be performed to assess the potential effects of farnesol *in vivo*. It must be emphasized that the toxicity of exogenously administrated farnesol on mice is negligible, as shown by Navarathna *et al*. [14]. Besides the observation of no significant gross changes in control and treated mice examined at necropsy, these authors verified that the oral (20 mM in water) or intraperitoneal (1 ml of 20 mM) administration of farnesol was harmless to mice, since there were no differences in weight or water ingestion between control and farnesol-treated animals.

In this sense, the antagonistic property of farnesol against *P. brasiliensis *cells is particularly interesting, since it could be further explored in order to evaluate its possible use as an antimicrobial agent.

## Competing interests

The authors declare that they have no competing interests.

## Authors' contributions

LD carried out all experiments and participated in the design of the study and the data analysis. SVB and SNB coordinated the electron microscopic study. CDSS and TMMDS participated in the design of the study. CK and ISP coordinated the study and critically evaluated the paper. ISP also received the financial support. All authors have read and approved the final manuscript.
